# Orthodontic shear bond strength and ultimate load tests of CAD/CAM produced artificial teeth

**DOI:** 10.1007/s00784-022-04676-7

**Published:** 2022-08-18

**Authors:** Christoph J. Roser, Thomas Rückschloß, Andreas Zenthöfer, Peter Rammelsberg, Christopher J. Lux, Stefan Rues

**Affiliations:** 1 Department of Orthodontics and Dentofacial Orthopedics, Heidelberg University Hospital, Im Neuenheimer Feld 400, 69120 Heidelberg, Germany; 2 Department of Oral and Maxillofacial Surgery, Heidelberg University Hospital, Im Neuenheimer Feld 400, 69120 Heidelberg, Germany; 3 Department of Prosthodontics, Heidelberg University Hospital, University of Heidelberg, Im Neuenheimer Feld 400, Heidelberg, Germany

**Keywords:** CAD/CAM, Dental, Bovine teeth, Artificial teeth, Shear bond strength, Ultimate load

## Abstract

**Objectives:**

To investigate whether artificial CAD/CAM processed (computer-aided design/manufacturing) teeth could be a feasible option for the production of dental in vitro models for biomechanical testing.

**Material and methods:**

Disks (*n* = 10 per group) made from two different CAD/CAM-materials, one fiber-reinforced composite (FRC; Trinia, Bicon) and one polymethylmethacrylate-based resin (PMMA; Telio CAD, Ivoclar Vivadent), as well as bovine teeth (*n* = 10), were tested for their shear bond strength (SBS) and scored according to the adhesive remnant index (ARI). In addition, CAD/CAM-manufactured lower incisor teeth were tested for their ultimate load (F_u_).

**Results:**

With regard to SBS, both PMMA (17.4 ± 2.2 MPa) and FRC (18.0 ± 2.4 MPa) disks showed no significant difference (*p* = 0.968) compared to bovine disks (18.0 ± 5.4 MPa). However, the samples differed with regard to their failure mode (PMMA: ARI 4, delamination failure; FRC: ARI 0 and bovine: ARI 1.6, both adhesive failure). With regard to F_u_, FRC-based teeth could withstand significantly higher loads (708 ± 126 N) than PMMA-based teeth (345 ± 109 N) (*p* < 0.01).

**Conclusion:**

Unlike PMMA-based teeth, teeth made from FRC showed sufficiently high fracture resistance and comparable SBS. Thus, FRC teeth could be a promising alternative for the production of dental in vitro models for orthodontic testing.

**Clinical relevance:**

CAD/CAM-processed teeth made from FRC enable the use of standardized geometry and constant material properties. Using FRC teeth in dental in vitro studies has therefore the potential to identify differences between various treatment options with rather small sample sizes, while remaining close to the clinical situation.

## Introduction 

When it comes to the establishment of new materials, devices, and methodologies, dental in vitro testing is of particular importance, as it helps to estimate study parameters for subsequent clinical investigations and thereby protects patients from unnecessary detrimental burden. However, constructing dental in vitro models is demanding, because of the limited availability of undamaged human extracted teeth. Moreover, because human teeth cannot usually be obtained from a single individual, in view of standardization, teeth with variable properties (in terms of geometry, size, enamel texture, etc.) hamper the production of a full dentition model which is comparable to the clinical situation. For shear bond strength (SBS) testing, bovine teeth of cattle aged between 2 and 5 years are accepted as substitutes for human teeth according to DIN 13990–1. Studies comparing the influence of different substrates on tensile or shear bond strength showed that the SBS of human and bovine teeth was similar [1–6]. However, due to their size, bovine teeth are not suitable for full dentition models. Generally spoken, metallic teeth provide bond strengths which are above those found for human teeth and stiffer than human teeth. Resin teeth have the disadvantage that they can withstand only rather small oblique forces and are too malleable. A rather new millable fiber-reinforced composite (FRC) seemed to be a good approach for overcoming these shortcomings. Therefore, in the present study, SBS and ultimate load (F_u_) tests were performed with the aim of comparing the results of samples made of FRC- or polymethylmethacrylate-based resin (PMMA). The working hypothesis was that both computer-aided design/manufacturing (CAD/CAM) materials show no difference in SBS in comparison to bovine teeth. Moreover, the present study investigated whether teeth made from both CAD/CAM materials have sufficient F_u_ values above physiological mastication force.

## Materials and methods

### Preparation of specimens for SBS testing

For the SBS tests, two groups (*n* = 10 per group) with disks cut (IsoMet High Speed Pro, Buehler, Uzwil, Switzerland) from both CAD/CAM materials, FRC (Trinia, Bicon; Boston, USA) and PMMA (Telio CAD, Ivoclar Vivadent. Schaan, Liechtenstein), and one group with bovine teeth (*n* = 10) were investigated. Bovine teeth were purchased from Rocholl GmbH (Eschelbronn, Germany). Storage after extraction and preparation of bovine samples were performed according to DIN 13990–1. After placement in a cylindric mold (25 mm in diameter), the disks and teeth were embedded in acrylic resin (Technovit 4071, Kulzer, Hanau, Germany). Specific attention was paid that the bonding surfaces were adjusted parallel to the subsequent SBS test direction. In a next step, PMMA and FRC disks were ground flat (#220 SiC paper; Tegramin25, Struers, Willich, Germany) and cleaned with ethanol in an ultrasonic cleaning device. For bonding preparation, PMMA and FRC disks were sandblasted (50-µm alumina particles, 1 bar) and conditioned with the appropriate primer (Table 1). Each of the bovine teeth was polished with pumice powder (50 g/40 g water) for each 3 s in the directions mesial-distal and occlusal-gingival and with a linen polishing disk (Erkodent, Pfalzgrafenweiler, Germany). Bovine teeth which still showed staining in the planned bonding area after this treatment were excluded from the investigation. Bovine teeth which were included in the investigation were acid etched with 37% phosphoric acid gel (Omni-Etch; Omnident, Rodgau, Germany) for 30 s and cleaned with water for another 30 s. Then, the respective primers were applied (Table 1) according to the manufacturer’s information. All preconditioned disks were complemented with composite pins (3 mm in diameter; Transbond XT; 3 M, Saint Paul, USA) using a silicone template. In order to guarantee continuous curing, composite was applied in two parts and light cured respectively (40 s; 460 nm; Smartlite focus; Dentsply Sirona, York, USA). After manufacturing, all samples for SBS testing were stored in distilled water at body temperature (37 ± 1 °C) for 24 h according to previous studies [7–9].

**Table 1:** List of relevant information of all materials used in the present study — *according to manufacturer’s information, PMMA (polymethylmethacrylate), FRC (fiber-reinforced composite)

Material	Brand name (manufacturer)	Primer for SBS test	Young’s modulus [GPa]	Tensile strength [MPa]
PMMA	Telio CAD (Ivoclar Vivadent)	Visio.Link (Bredent)	3.2^*^	130^*^
FRC	Trinia (Bicon)	Ceraresin Bond (Shofu)	18.8^*^	Parallel: 393^*^ Perpendicular: 169^*^
Bovine tooth	-	Optibond FL primer and adhesive (Kerr)	Enamel: 72 ± 6 [10] Dentine: 16.1 ± 1.4 [11]	E: 30.5 ± 3.3 [12] D: 85.4 ± 3.0 [12]

### SBS measurements

**Fig. 1:**
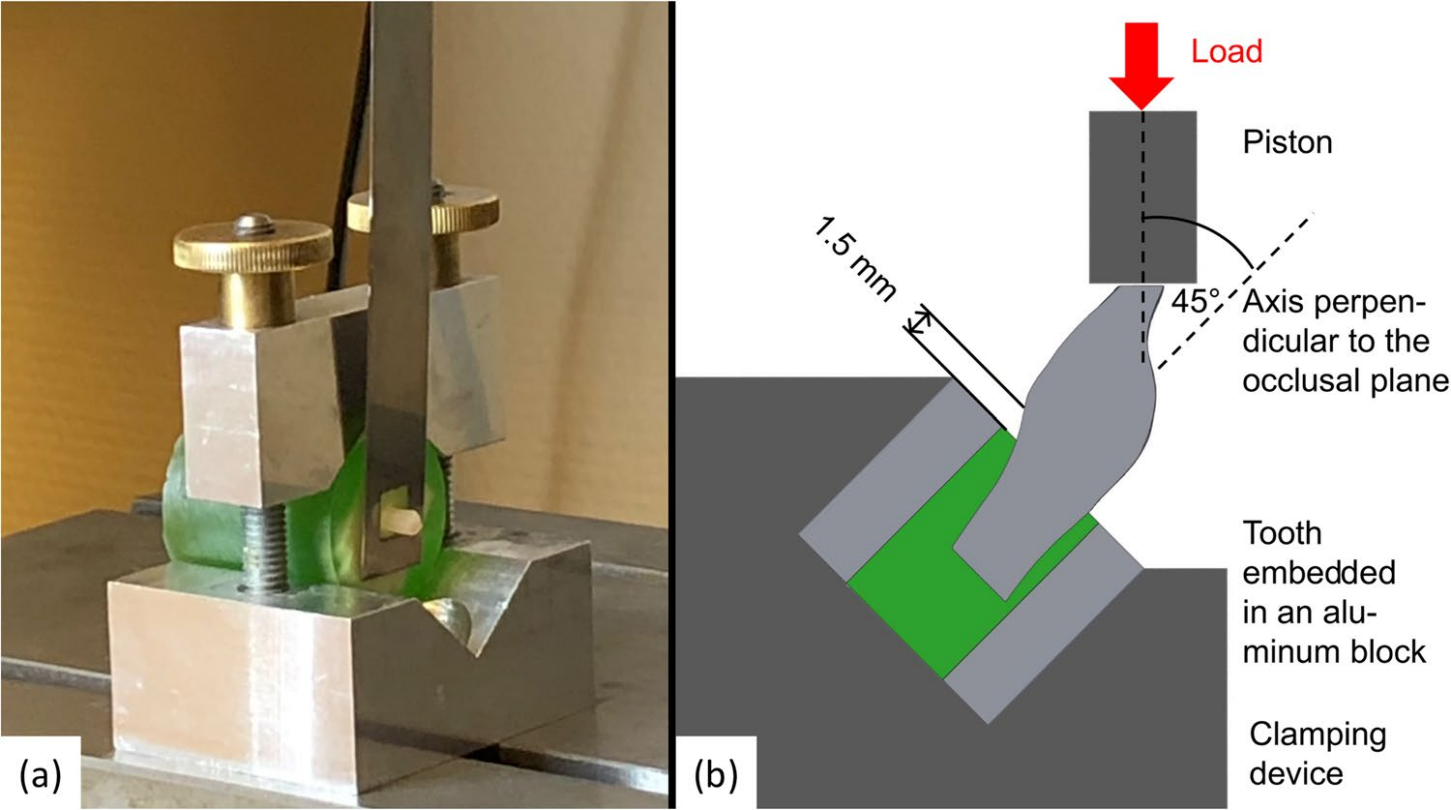
Setup of shear bond strength (SBS; **a**) and fracture (Fu; **b**) tests — SBS tests were performed according to 13,990–1 on both CAD/CAM materials and bovine teeth. Moreover, Fu tests were performed on teeth which were previously manufactured from both CAD/CAM-materials. Load application was tilted by 45° in the sagittal plane in order to simulate a particular critical case

SBS testing was carried out in a universal testing device (Z005, Zwick/Roell, Ulm, Germany; Fig. 1a) according to DIN 13990–1. After SBS testing, surfaces of all disks/teeth were investigated for their failure mode using a digital microscope (Smartzoom 5, Zeiss, Wetzlar, Germany) at × 64 magnification and scored with the adhesive remnant index (ARI) according to [10] (Table 2).

**Table 2:** Modified version of the adhesive remnant index (ARI) according to Ju et al. [13]

ARI	Failure
0	No adhesive left on the tooth/disk
1	< 50% adhesive left on the tooth/disk
2	> 50% adhesive left on the tooth/disk
3	All adhesive left on the tooth/disk
4	Disk/enamel fracture

### F_u _measurements

Tests of *F*_*u*_ were performed according to a previous study [14] on artificial lower incisor teeth, which had previously been digitally designed and milled from each CAD/CAM material (Fig. 1b). FRC teeth were nested in such a way that the mesial-distal direction was oriented vertically, i.e., perpendicularly with respect to the glass fiber sheets. The teeth were embedded (Technovit 4071) in aluminum blocks (with the surface of the blocs parallel to the occlusal plane) with the resin surface placed 2 mm below the cement-enamel junction. After production, all teeth for *F*_*u*_ were stored in water for 30 ± 1 days in distilled water at body temperature (37 ± 1 °C). Then, after placement of the samples with a tilt of 45°, loading took place in a vertical direction with a crosshead speed of 5 mm/min. The test ended when either a drop of > 80% of the maximum force took place or vertical displacement reached 2 mm.

### Sample size calculation

Because clinically meaningful effect sizes are not available from the literature, prior to our main investigation, we performed tests with three specimens of each group and found shear bond strength values of 18.3 ± 3.19 MPa (FRC), 16.68 ± 1.2 MPa (PMMA), 17.35 ± 3.7 MPa (bovine). These results for bovine teeth were similar to previous studies [7, 15] and fell in the middle of the range of published results (7.8 ± 6.2 and 30.7 ± 5.7 MPa), which was shown in a recent review [16]. Therefore, we considered our results to be valid for sample size calculation. Since the standard on which our tests are based does not provide any definitive shear bond strength limits, we decided to use the clinically necessary shear forces for the enamel-bracket bond as a basis. Thus, for effect size determination, we considered a minimum shear bond strength of 6–8 MPa to be clinically relevant, as it is widely accepted [17, 18]. Based on the results obtained for bovine teeth in of our pilot study (17.35 ± 3.7 MPa), we determined 11 MPa as the effect size and calculated the sample size as follows: significance level: 5%, statistical power: 0.8, effect size: 11 MPa, standard deviation: 4 MPa which yielded a sample size of 4. Therefore, we considered a sample size of *n* = 10 to be sufficient for valid results.

### Statistical analysis

Statistical analysis was performed using SPSS 27 (IBM, Endicott, USA). Since all groups were independent in both tests, SBS data of bovine, PMMA, and FRC samples were analyzed using Kruskal–Wallis test and the *F*_*u*_ data of PMMA and FRC teeth were analyzed using Mann–Whitney *U*-test. Local statistical significance was assumed at alpha = 0.05.

## Results

### SBS measurement and corresponding ARI

**Fig. 2:**
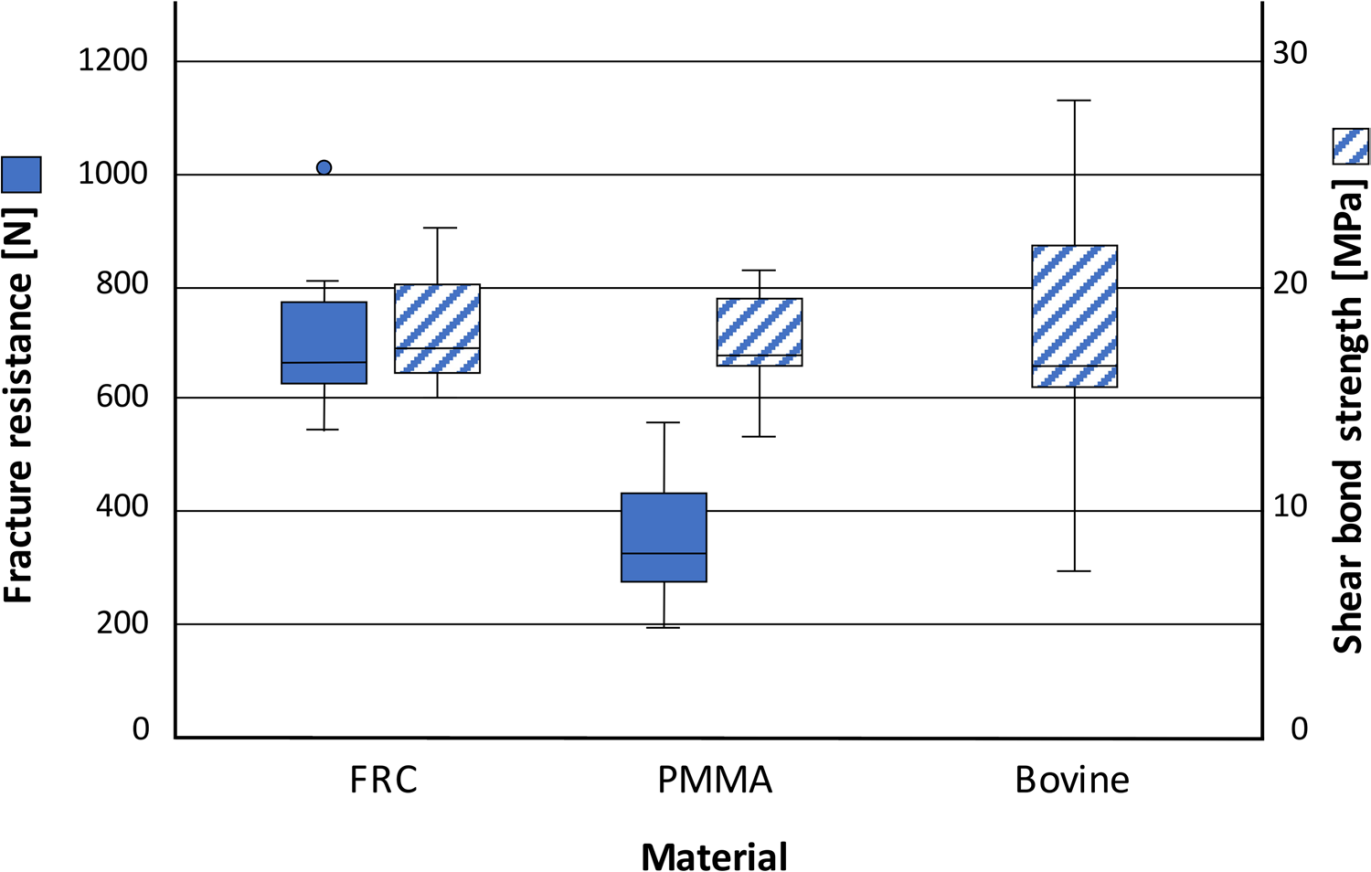
Results of ultimate load (F_u_) and shear bond strength (SBS) tests — While all three groups showed comparable results in SBS, there was higher variance in the results of bovine teeth. FRC (fiber-reinforced composite), PMMA (polymethacrylate)

**Fig. 3:**
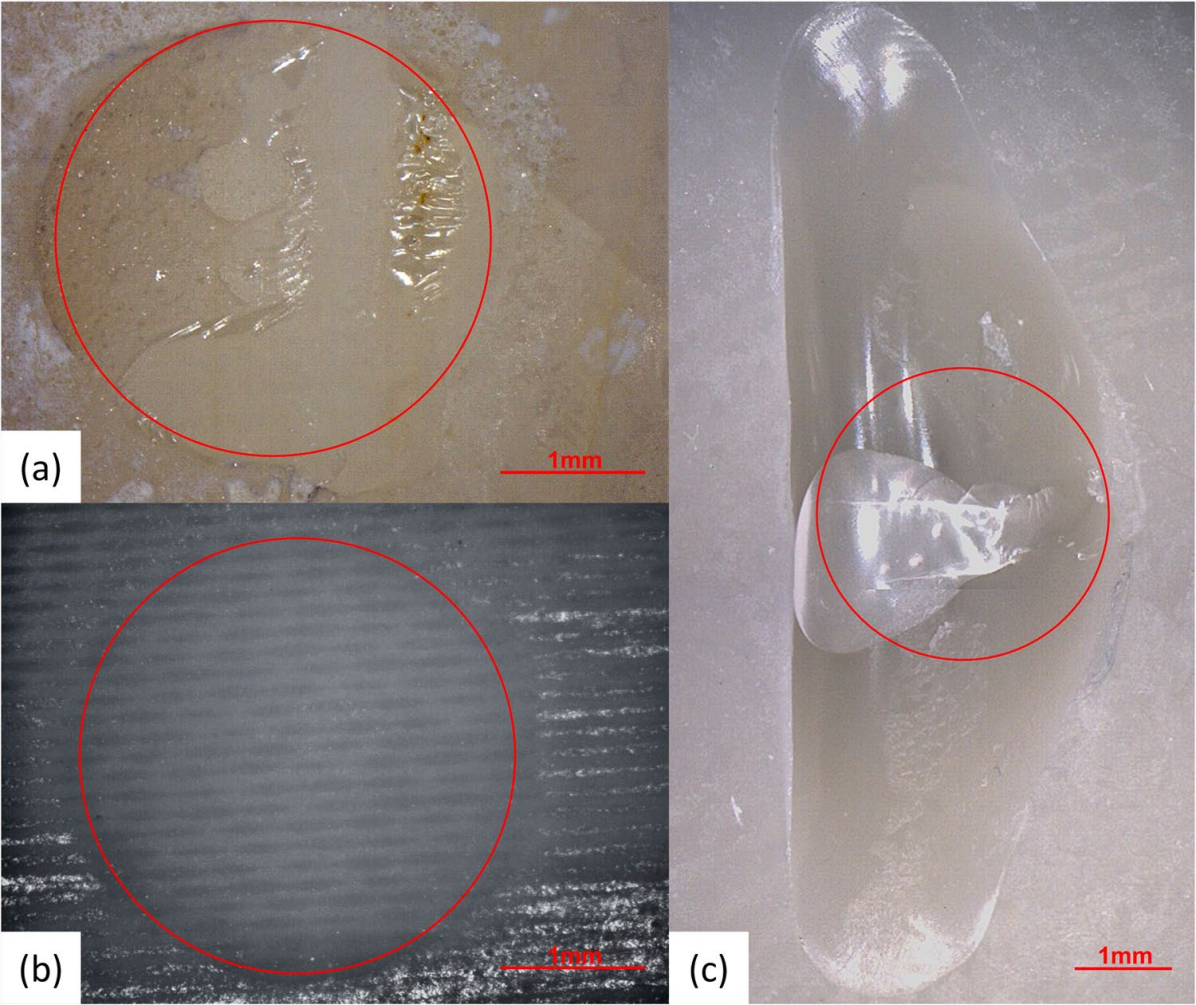
Surfaces after shear bond strength tests — while both bovine teeth (mean adhesive remnant index (ARI): 1.6; **a**) and fiber-reinforced samples (all ARI: 0; **b**) showed adhesive failure, all PMMA samples failed cohesively within the substrate (ARI: 4; **c**). In this way, the cohesive failure surface of PMMA samples exceeded the bonding area (red circle)

Test results are presented in Table 3 and Fig. 2. Mean SBS values for both PMMA (17.4 ± 2.2 MPa) and FRC (18.0 ± 2.4 MPa) samples showed no significant difference (*p* = 0.968) compared to bovine samples (18.0 ± 5.4 MPa). However, the samples differed with regard to their fracture mode. All FRC samples showed adhesive failure with an ARI of 0 (Fig. 3b). In contrast, all PMMA samples fractured cohesively within the substrate (ARI: 4; Fig. 3c). Within the bovine teeth, all teeth showed adhesive failure but differed regarding the ARI. Two teeth showed ARI 0, two teeth showed ARI 1, four teeth showed ARI 2, and two teeth showed ARI 3. Mean ARI for bovine samples was 1.6 (Fig. 3a).

**Table 3:** Results of shear bond strength (SBS) and ultimate load (F_u_) tests — all samples showed comparable results for SBS. However, the samples differed with regard to their fracture mode and their corresponding adhesive remnant index (ARI). With regard to F_u_, the teeth made from FRC showed significantly higher (*p* < 0.01) results compared to the PMMA teeth

	SBS [MPa]				Fracture mode (ARI)	F_u_ [N]			
Material	Mean	SD	Min	Max		Mean	SD	Min	Max
FRC	18.0	2.4	15.0	22.3	Adhesive (all ARI**:** 0)	708	126	547	1016
PMMA	17.4	2.2	13.3	20.7	Cohesive (all ARI**:** 4)	345	109	191	558
Bovine	18.0	5.4	7.2	23.5	Adhesive (ARI 0**:** 2; ARI 1**:** 2; ARI 2**:** 4; ARI 3**:** 2; ARI 4**:** 0; mean ARI**:** 1.6)

### F_u _measurement

With regard to *F*_*u*_, FRC teeth reached mean higher fracture resistances of *F*_*u*_ = 708.2 ± 125.8 N in contrast to PMMA teeth with *F*_*u*_ = 344.8 ± 108.8 N. Accordingly, *F*_*u*_ ranged between 547 and 1016 N for FRC and 191 and 558 N for PMMA, respectively. This difference was highly significant (*p* < 0.001).

## Discussion

The working hypothesis was confirmed, because FRC and PMMA CAD/CAM materials showed no significant difference in SBS compared to bovine teeth with mean SBS values in all test groups between 17 and 18 MPa.

However, PMMA samples were the only ones which showed cohesive fracture within the substrate during SBS testing (all ARI: 4). Hence, PMMA-based CAD/CAM materials might be unsuitable to replace natural teeth in in vitro SBS tests for which the outcome is affected by the adhesive connection between teeth and orthodontic device or dental restoration. In contrast, all FRC samples were associated with an adhesive failure mode (all ARI: 0). Likewise, bovine teeth showed a solely adhesive failure but differed slightly with respect to ARI (mean ARI: 1.6). Therefore, our results, which were in line with previous studies on human teeth (ARI: 1.8; [19]) and bovine teeth (ARI: 2; [7]), demonstrate that, among the teeth made of FRC, a similar fracture mode and fracture strength compared to bovine teeth can be achieved. Nevertheless, when precisely examining the bonding area, different ARI values might have to be taken in account. However, this has to be investigated in further studies with different primers and bonding systems.

Furthermore, both FRC and PMMA lower incisor teeth could withstand mean *F*_*u*_ ranging above the maximum physiological mastication force, which reaches a maximum of 270 N in the axial direction [20–23] and 200 N when tilted by 45° in the sagittal plane [24]. It is important to note here that *F*_*u*_ tests were performed tilted by 45° in the sagittal plane in order to simulate a particular critical load case. As long as the load is applied in the tooth axis, phantom teeth made of any restorative material will generally not fracture, since compressive strength values are far above the tensile material strength values. However, since for many treatment-concepts loads tilted with respect to the tooth axis state a more critical case compared with axial loading, phantom teeth are required to withstand in vitro simulations with tilted force application. The initial *F*_*u*_ values of all incisor teeth except for one (PMMA: *F*_*u*,min_ = 191 N, see Table 2) were above 200 N. However, nearly half of the PMMA teeth showed fracture values of *F*_*u*_ < 300 N. Therefore, longer periods of water storage or procedures like chewing simulation or thermocycling prior to *F*_*u*_ tests might be critical for teeth made of PMMA. If the material’s strength decreases due to aging, the failure rate of phantom teeth below the 200 N threshold will increase and FRC teeth, on the other hand, had a much higher *F*_*u*_ (*p* < 0.001) which exceeded 550 N. With such a safety margin, FRC teeth should not be affected by artificial aging in chewing simulations, which are typically carried out with force magnitudes ranging between 50 and 100 N. In addition, compared to PMMA, the Young’s modulus of FRC is closer to that of either enamel or dentine (Table 1).

Moreover, the present study demonstrated that SBS tests on bovine teeth, the results of which were in line with previous studies [6, 25, 26], were combined with a particular high standard abbreviation which was about twice as high compared to both CAD/CAM materials. Similar to even higher standard abbreviation values were shown in previous studies, including those with a higher sample size [3, 7, 27–31]. This might be due to the fact that bovine enamel is not as standardized as industrially produced restorative materials, mostly because of the difference in substrate morphology, i.e., perfectly flat disks with FRC and PMMA in contrast to rather planar tooth surfaces with bovine teeth. Moreover, this might explain the different results in studies which tested SBS on bovine teeth before [16] and may reflect the ongoing discussion on the usage of bovine teeth as an alternative for human teeth in SBS testing [32]. In contrast, using artificial teeth made from FRC might provide testing under the highest standardization possibilities and therefore allow for easier comparison with other studies. Furthermore, in contrast to human or bovine teeth which are often hard to obtain, CAD/CAM teeth can be easily created in different geometries for any kind of in vitro situation required. In addition, changes within one tooth design can be easily implanted digitally and designs which are made once can be easily manufactured again by use of a 3D printer.

To the best of our knowledge, this is the first investigation to evaluate the suitability of artificial teeth for in vitro testing. We compared artificial teeth with bovine teeth with respect to SBS while withstanding physiological mastication forces. Our study intended to validate artificial teeth for further investigations as for instance in vitro tests on different orthodontic materials and designs such as brackets, fixed retainers, or on prosthodontic adhesive restorations. Comparison between our newly introduced concept and others is limited, since previous studies with artificial teeth solely concentrated on the suitability of different materials for prosthodontic dentures with a focus on maximal stability to the acrylic base [33–38], sufficient optical properties and color stability [39], or evaluated concepts for educational purposes or validation of endodontic procedures [40–43].

When interpreting the results of the present study, several limitations have to be considered. For our investigation, we chose Transbond XT as the adhesive of choice, because it is widely accepted as the orthodontic gold standard adhesive [9, 44, 45]. Using FRC teeth with other adhesives might lead to different results. This is also the case for our standardized aging protocol for SBS testing, which stood in agreement with previous studies [7–9], including 24 h of water storage in 37 ± 1 °C water. Differences in the water storage period might affect SBS values. Therefore, both different periods of water storage and using other adhesives have to be investigated by further studies.

To this end, this is the first study which validated artificial FRC CAD/CAM teeth as an alternative to bovine teeth in in vitro testing. Using artificial FRC CAD/CAM teeth might facilitate the easier and more standardized production of dental in vitro models, simplifying the testing of different devices, materials, and methodologies in an in vitro setting in order to protect patients from unnecessary detrimental effects in subsequent clinical studies.

## Conclusions

Within the limitations of an in vitro setting, the following conclusion can be drawn based on the results of the present study:

- Artificial teeth made from FRC-based CAD/CAM materials show a sufficiently high *F*_*u*_ and comparable SBS results compared to bovine teeth and might therefore represent a feasible option for the construction of dental models for biomechanical in vitro testing of different devices and restorations.
- Artificial teeth made from PMMA-based CAD/CAM materials also show SBS values comparable to bovine teeth but exhibit inacceptable results in *F*_*u*_ and might therefore not be suitable for in vitro testing.
